# A Transgender Health Information Resource: Participatory Design Study

**DOI:** 10.2196/42382

**Published:** 2023-06-15

**Authors:** Brad Morse, Andrey Soares, Bethany M Kwan, Marvyn Allen, Rita S Lee, Kristen Desanto, Brooke Dorsey Holliman, Kate Ytell, Lisa M Schilling

**Affiliations:** 1 Division of General Internal Medicine, Department of Medicine University of Colorado Anschutz Medical Campus Aurora, CO United States; 2 Adult and Child Center for Outcomes Research and Delivery Science University of Colorado Anschutz Medical Campus Aurora, CO United States; 3 Department of Emergency Medicine University of Colorado Anschutz Medical Campus Aurora, CO United States; 4 One Colorado Denver, CO United States; 5 Strauss Health Sciences Library University of Colorado Anschutz Medical Campus Aurora, CO United States; 6 Department of Family Medicine University of Colorado Anschutz Medical Campus Aurora, CO United States; 7 Elevance Health Denver, CO United States

**Keywords:** lesbian, gay, bisexual, transgender, and queer, LGBTQ, transgender, mobile app, health information, participatory design, agile development, mobile phone

## Abstract

**Background:**

Despite the abundance of health information on the internet for people who identify as transgender and gender diverse (TGD), much of the content used is found on social media channels, requiring individuals to vet the information for relevance and quality.

**Objective:**

We developed a prototype transgender health information resource (TGHIR) delivered via a mobile app to provide credible health and wellness information for people who are TGD.

**Methods:**

We partnered with the TGD community and used a participatory design approach that included focus groups and co-design sessions to identify users’ needs and priorities. We used the Agile software development methodology to build the prototype. A medical librarian and physicians with expertise in transgender health curated a set of 97 information resources that constituted the foundational content of the prototype. To evaluate the prototype TGHIR app, we assessed the app with test users, using a single item from the System Usability Scale to assess feature usability, cognitive walk-throughs, and the user version of the Mobile Application Rating Scale to evaluate the app’s objective and subjective quality.

**Results:**

A total of 13 people who identified as TGD or TGD allies rated their satisfaction with 9 of 10 (90%) app features as *good* to *excellent*, and 1 (10%) of the features—the ability to filter to narrow TGHIR resources—was rated as *okay*. The overall quality score on the user version of the Mobile Application Rating Scale was 4.25 out of 5 after 4 weeks of use, indicating a good-quality mobile app. The information subscore received the highest rating, at 4.75 out of 5.

**Conclusions:**

Community partnership and participatory design were effective in the development of the TGHIR app, resulting in an information resource app with satisfactory features and overall high-quality ratings. Test users felt that the TGHIR app would be helpful for people who are TGD and their care partners.

## Introduction

### Transgender Health Disparities and Inequities

The term *transgender and gender diverse* (TGD) refers to individuals whose gender identity does not align with their sex assigned at birth and includes individuals who identify as transgender, gender nonbinary, gender diverse, or gender-fluid [1]. The term *transgender* can be viewed in contrast to the term *cisgender*, which is used to describe those whose gender identity aligns with their sex assigned at birth.

The social determinants of health and structural stigma create health barriers for the TGD population. For example, 29% of the respondents of the 2015 US Transgender Survey lived in poverty compared with 12% of the US population [2]. In addition, the unemployment rate of respondents was 15% compared with 5% in the general US population [2]. As a result, the TGD community experiences many barriers to achieving health and well-being. Relative to cisgender individuals, transgender individuals experience disparities and inequities in all aspects of health (ie, mental, physical, emotional, and social), including poor overall health status, access to quality health care, and mental health, as well as an increased risk of substance use disorder, myocardial infarction, and sexual and reproductive health concerns [3-7]. Transgender individuals commonly experience trauma, abuse, and violence throughout their life span, leading to chronic stress [8,9]. Persons who are TGD experience many social determinants linked to poor health, including a lack of stable income and quality housing [10-12]. Stigma [13,14], ignorance [15], and discrimination contribute to poor health care access and poor care quality. Lack of social support, ranging from social exclusion and marginalization to poor social and family relationships, including rejection and family violence, contributes to stress and mental illness, including suicide [16]. Persons who are TGD are often poorly treated in the health care system and have difficulty finding TGD-competent and knowledgeable providers [15]. As a result, persons who are TGD may avoid routine care and are less likely to receive preventive services [17], less adherent to life-saving therapy [18-20], and more likely to experience denial of services from primary care to end-of-life care [21,22] and to be uninsured or have public insurance than persons who are cisgender [23]. In 2015, the American College of Physicians advocated for the creation of policies that would advance health equity among the lesbian, gay, bisexual, transgender, queer or questioning (LGBTQ) community—including the need for ongoing research on best practices for equitable health care [24]. Complicating the need for TGD-competent care is the fact that persons who are TGD experience unique needs related to gender-affirming health care as well as general health care and that their needs evolve across their life span, from youth and adolescence through midlife and late life [13,25-27].

### Health Information Needs of Persons Who Are TGD

The existing literature shows that persons who are TGD and their caregivers most often seek information to (1) explore gender identity and *coming out*, (2) fill health and medical knowledge gaps, (3) seek support networks, (4) find TGD-competent providers, (5) find legal advice, and (6) find advocacy/political advice [28,29]. Legal issues important to health and well-being include protections, such as health insurance discrimination, and policies and procedures for changing gender information on legal forms of identification such as a driver’s license [30]. Persons who are TGD also seek information to build skills for communication with health care providers [25,31] and information regarding strategies and counseling services to build resilience, improve body image, and contend with other stressors [32]. In addition, the TGD community needs specific medical and health information regarding gender-affirming care, including surgery, mental health support, and hormone treatments and their impact [28,29,33-37].

Barriers to finding relevant health information include (1) a general lack of TGD health information [35], (2) not knowing the terms to use when searching [35], (3) often finding hateful content and misinformation [34], and (4) identifying credible and reliable sources of health and medical information. The TGD community relies heavily on the internet for all types of information [29]. Having access to credible sources of health information could serve to balance the increasing number of articles or posts that contain misinformation or are outdated [38,39]. Evans et al [28] highlighted the need for credible and trustworthy web-based content. Relevant TGD health information should be easy to access and broadly meet the diverse needs of the TGD community [34].

### Transgender Health Information Sources and Accessibility

A 2012 review identified and categorized several websites created for the TGD community [40]. Within the health domain, HIV was the focus of most of the websites (n=17), followed by gender-affirming surgery (n=8), mental health (n=2), primary care (n=1), and sexual health (n=1). Over the last decade, with support from the National Library of Medicine, public and health sciences libraries have focused on cataloging information resources for the TGD community, including establishing transgender resource library collections and reference services [25,26,29,41,42]. The Lesbian, Gay, Bisexual, and Transgender Health Resources Guide of the University of Colorado Strauss Health Sciences Library [43] is one such collection.

Owing to the phenomenon of mobile apps for dating and social networking, their use among the LGBTQ community has been studied extensively to assess users’ risk of HIV and sexually transmitted infections [44-47]. A recent study by Akinola et al [36] assessed the barriers and facilitators for Black transgender women to the use of mobile app technology for HIV self-testing and remote research participation. Reported facilitators included being more engaged and having increased self-agency, whereas barriers included inconsistent access to the internet and smartphones. Radix et al [48] concluded that the use of health ITs (HITs) provides opportunities to improve the quality of care for TGD individuals. Not only can HIT solutions be designed to offer education and support addressing the social determinants of health, but the community also favors these solutions [48].

### Objectives

Our objective was to use participatory design methods to design a health information resource to support persons who are TGD in finding and using credible health information prioritized according to their needs. We selected a delivery method known to combat the disparities of the digital divide and known to be used by the LGBTQ community already—mobile smartphones [44-47,49,50].

## Methods

### Overview

**Figure 1:**
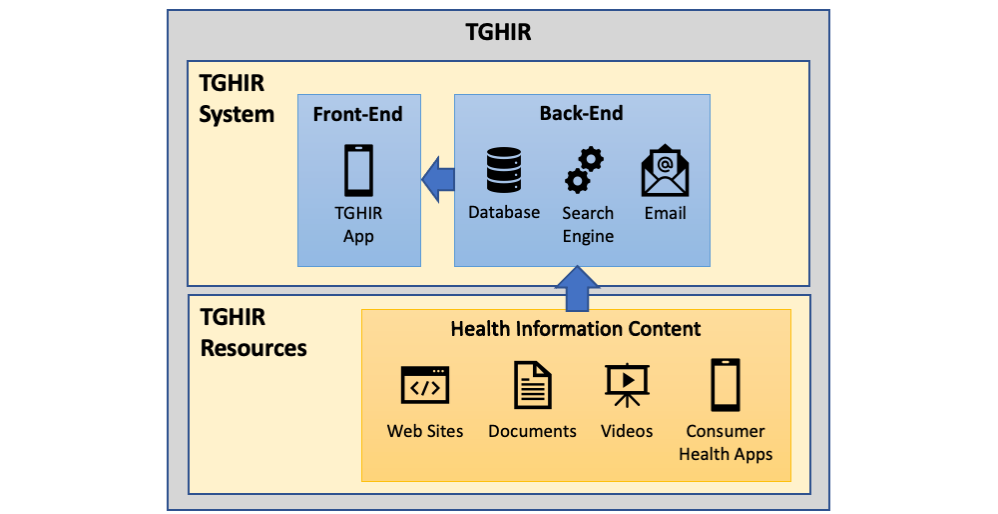
Overview of the transgender health information resource (TGHIR) components.

This section describes the design and development of our transgender health information resource (TGHIR [*tigger*]) [51,52]. The TGHIR platform consists of three main components (Figure 1): (1) curated health and wellness information content (henceforth, *TGHIR resources*), such as websites, documents, videos, and other consumer health apps; (2) a back-end system, including a database to store user data and TGHIR resources, a search engine, and a communication platform that allows users to send messages to the development team (eg, comments/feedback, suggestions of new content, and reports of offensive or inaccurate content); and (3) a front-end mobile app (henceforth, TGHIR app) that allows users to create accounts, search for information, and access/view the curated TGHIR resources. We did not create any of the TGHIR resources and, instead, incorporated links to freely available resources that can be accessed via the internet. The TGHIR resources were used as input to populate the database and as *seeds* to create the search engine.

### Study Design

**Figure 2:**
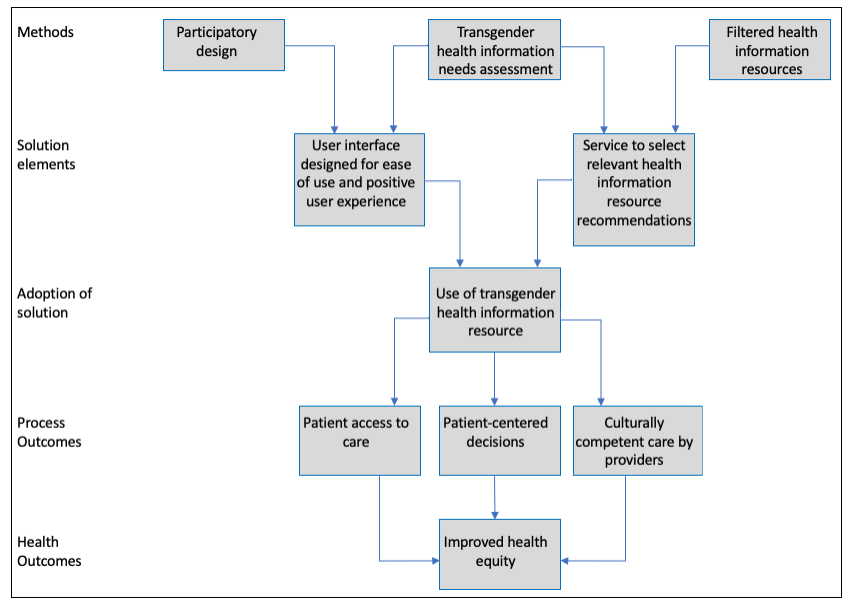
Conceptual framework for the design and development of the transgender health information resource.

We based the design and development of the TGHIR app on a conceptual framework (Figure 2) that recognized the importance of participatory design, which is a form of user-centered design that focuses on designing *with* end users and not merely *for* end users [53]. Participatory design prioritizes users and allows for their direct participation in the design process through decision-making, going beyond the role of users as consultants. Direct involvement in the entire design process often leads to greater satisfaction with both the process and the outcome [51,52]. Participatory design also represents a key strategy for designing for dissemination, sustainability, and equity, attending to potential factors that may influence widespread adoption and equitable access to the TGHIR app [54].

**Figure 3:**
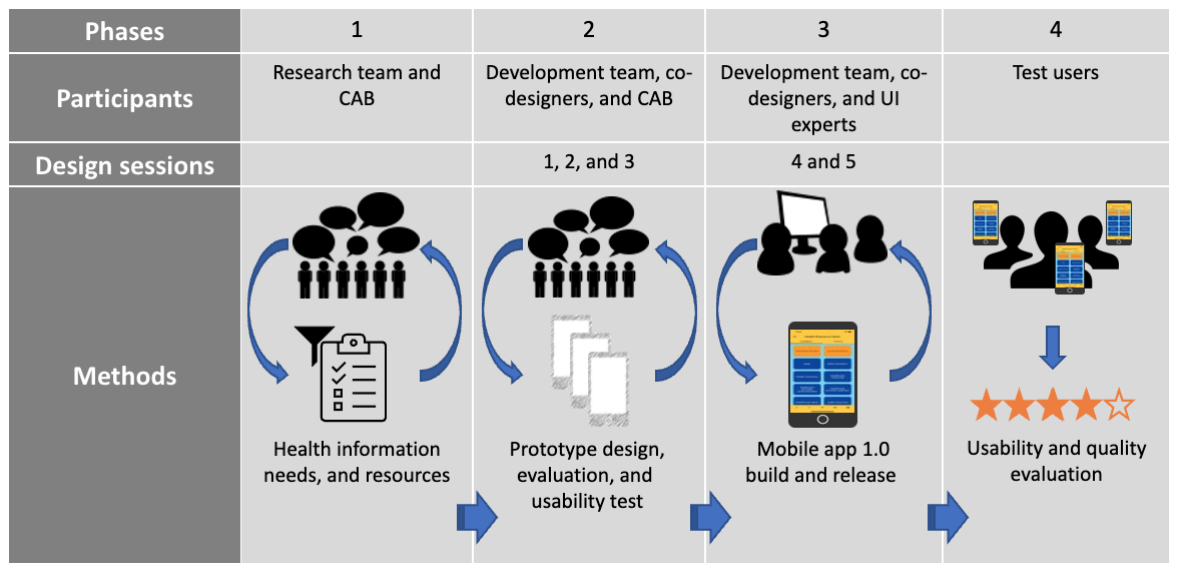
Phases used to design and develop the transgender health information resource. CAB: Community Advisory Board; UI: user interface.

We applied the basic stages of participatory design by Spinuzzi [55]—stage 1: initial exploration of work; stage 2: discovery processes; and stage 3: prototyping—and implemented a participatory design process described by Schnall et al [56]. We applied the 4-phase approach by Schnall et al [56] to the design and evaluation of the TGHIR app (Figure 3). In this paper, we sequentially summarize the methods and results by phase as the results from subsequent phases inform later phases.

### Ethics Approval

The project was approved as exempt human participant research by the Colorado Multiple Institutional Review Board (protocol 19-1562).

### Collaborators

#### Overview

Collaborators involved in the development, design, and evaluation of the TGHIR included our research team; an LGBTQ community-based advocacy organization (One Colorado); our Community Advisory Board (CAB); and research participants, including focus group (FG) participants, design session co-designers, HIT experts, and test users. One Colorado is a leading advocacy organization in Colorado dedicated to advancing equality for LGBTQ Coloradans and their families [57]. The research team consisted of individuals with clinical, informatics, library/information science, dissemination and implementation science, and health service research expertise.

In partnership with One Colorado, we established and engaged a CAB. The CAB included 20 individuals. They were 60% (12/20) community members and 40% (8/20) research team members. CAB community members included 7 people who identified as TGD; 4 parents of TGD adolescents; and 3 clinicians with expertise in TGD health care, including primary care, surgery, and mental health. Some CAB members belonged to more than one category. We interviewed everyone to confirm their interest in and commitment to the project. The CAB was engaged throughout all phases of the design and development process.

#### Research Participant Recruitment

In collaboration with One Colorado, research participants were recruited using a series of Facebook recruitment posts in private transgender groups and physical fliers posted at the Integrated Transgender Clinic [58] at the University of Colorado Anschutz Medical Campus. People expressing interest in the project first met via Zoom (Zoom Video Communications) [59], a communication and collaboration platform, with the project manager, who also co-led the design sessions, to confirm interest in and fit for the project. Our goal was to recruit those who were TGD or TGD allies and who wanted to contribute to a participatory design process to design a TGD health information resource.

Overall, 42 TGD individuals responded to the recruitment advertisement, and 32 (76%) participated in phases 1 to 3. A total of 10 individuals were not eligible: 2 (20%) because of gender identity and age and 8 (80%) because of scheduling conflicts. A total of 81% (26/32) participated in an FG and 59% (19/32) participated in one or two design sessions, including 13 that attended both an FG and a design session. All 32 of the participants self-identified as either transgender, non-binary, gender queer, or other TGD category. The race and ethnicity reported by participants was mainly White (21/32, 66%) and other categories (11/32, 34%) included Hispanic, African American, Asian, Native American, and multiple races. Most participants were between 18 to 40 years of age, 72%, (23/32). In total, 2 HIT experts in usability were recruited from our medical center to participate in phase 3. A total of 13 test users not involved in phases 1 to 3 were recruited for phase 4 in the aforementioned manner. In total, 92% (12/13) of the test users identified as either transgender, nonbinary, or genderqueer, Due to low sample size we cannot report race, ethnicity or age ranges.

### Phase 1: Health Information Needs Assessment

Phase 1 was dedicated to establishing a relationship between the CAB and the research team, eliciting CAB member insights into the proposed methods, and identifying TGD-related health information needs and sources.

#### CAB Engagement

##### Overview

The project started with a 4-hour kickoff meeting with the CAB and the research team using Liberating Structures [60], which are meeting strategies and structures that replace traditional top-down meeting practices with whole-group interactions. We used the Liberating Structures Purpose-to-Practice exercise to generate shared purpose, principles, participants, structure, and practices, which helped define the research team’s and the CAB’s responsibilities and approach to the work. The CAB also reviewed a draft of the FG guide and recruitment fliers at the CAB kickoff meeting.

##### CAB Kickoff Insights

The CAB recommended edits to the FG materials and overall approach, including recommendations on (1) term use (eg, *transgender and gender diverse* rather than *transgender and nonbinary*) and (2) specific health topics (eg, mental health support and finding clinicians) that we should inquire about in the FGs. The CAB also ensured that we understood the history of challenges and dissatisfaction that the TGD community has had with the health care system in general. This included the challenges that the TGD community faces in finding clinically (ie, knowledge of evidence-based health care for TGD people) and culturally (ie, *trans-friendly*) competent care. The CAB stressed that a common problem in the TGD community is that health care professionals frequently attribute all medical conditions to being TGD and that TGD individuals must manage the same general health and medical needs as the cisgender community (eg, cancer prevention and broken bones). These insights not only improved our FG materials but also allowed us to be sensitive to the frustrations the community has with health care providers and systems, which to them the research team represented.

#### FG Engagement

##### Overview

We conducted 4 FGs with 26 participants (n=8, 31% in FG 1; n=6, 23% in FG 2; n=6, 23% in FG 3; and n=6, 23% in FG 4) following a phenomenological approach to design, conduct, and qualitatively analyze a TGD health information needs assessment [61]. We developed a semistructured FG guide using essential questions [61] to understand health information–seeking behavior (ie, how and where) and the types of health information sought (ie, what). We conducted web-based FGs using Zoom. FGs were recorded and professionally transcribed for analysis. We performed rapid analysis [62] of the transcripts to allow us to quickly use the information in the next resource information app development phase.

##### Results: FG

The FGs ultimately provided limited information about the specific types of health information needed by the TGD community as the FG participants organically oriented their discussions to the need for clinically and culturally competent care. However, 2 key insights did emerge from FG analyses. First, participants reported that their health care providers often did not have answers to TGD-specific health questions and advised them to seek information themselves using the internet. Second, many participants indicated that they relied heavily on peer-to-peer social media (eg, Reddit and private Facebook groups) for health information. However, this information was often felt to be difficult to interpret in the context of their specific gender identity and required vetting on their own. Taken together, these insights suggested that there was an unmet need regarding finding credible, personally relevant health information, and thus, an app such as the TGHIR app could be useful.

#### Health Information Source Identification and Curation

##### Overview

Additional strategies for identifying the types of health information often sought by TGD individuals and the corresponding sources of that information included performing literature and internet searches and seeking input from the CAB. CAB members emphasized the need for information about seeking health insurance, hormone therapy, and legal resources (eg, updating driver’s licenses and permission to be at the bedside of a hospitalized partner) and locating competent clinicians. The CAB also emphasized that the information resources should be sensitive to terms and stigmatizing language, accessibility (disability and non-English language), and inclusivity. In parallel, the research team’s medical librarian (KD) performed a literature search on a medical bibliographic database (ie, PubMed) for research articles on the health information–seeking behavior of TGD people. This process identified the categories of information most frequently searched on the web (eg, hormone therapy, health insurance, mental health support, and surgery options) [63-66]. Next, the librarian performed a Google search to identify health information resources freely available to the public in each of these categories. A set of search terms (Multimedia Appendix 1) was developed based on published terminology to identify LGBTQ-related information [67]. CAB members reviewed and provided insights on the list of health information resources, specifically focusing on issues of credibility and inclusivity. These recommendations were aligned with the research team, and the CAB provided critical suggestions on how to carry them out. It was deemed important that users of the TGHIR app understand how to assess web-based health information for credibility as it would not be sustainable to have a medical librarian or health professional review every potential resource. We asked the CAB to review available layperson credibility assessment tools (Multimedia Appendix 2) that could be incorporated into the app and help users make judgments for themselves. The CAB agreed that the simpler of the tools, *Trust it or Trash it* [68], would work best for determining credibility. The CAB asserted that this tool was informal, the format was easy to follow, and it was more accessible than a MEDLINE tutorial on how to evaluate health information found on the internet [69]. The CAB also wanted to ensure that the app was *inclusive* regarding the broad range of gender-diverse identities. For them, this meant using terms acceptable to the TGD community and including a broad range of information for TGD persons across their life span. CAB members suggested search tags and category labels (described in the following section) to ensure the use of culturally acceptable and commonly used terms to find resources within the app. The librarian and clinician research team members reviewed the initial sources for credibility before finalizing the resource list, tags, and category labels.

##### Results: Health Information Curation

A total of 97 credible health information resources in 16 topical categories (Multimedia Appendix 3) were identified and cataloged for this project. The list of resources and categories was often revised and updated as new resources were suggested or discovered throughout the project. The medical librarian ensured that all the web links were active and provided access to the expected content. Each information resource was cataloged regarding name/title, authoring organization, topic (eg, health, mental health and social support, or legal and financial), potential search tags, and category (eg, surgery and health care rights) for storage in the TGHIR database.

### Phase 2: Prototype Design, Evaluation, and Usability Test

#### Overview

In phase 2, we identified and prioritized features and designed prototypes (mock-up screens) using Justinmind [70]. In a series of web-based sessions, participatory co-designers considered *how* they would want to access and use TGD health information resources via the TGHIR app. Table 1 provides an overview of the 4 web-based design sessions that occurred in phases 2 (sessions 1-3) and 3 (session 4), including the number of participants and the primary methods used.

**Table 1:** Overview of the design sessions conducted in phases 2 and 3.

Session	Research participants, N	Goals/tasks	Design approach
1: feature requirement exploration	4	Exploration of mobile app features liked and used and value proposition generation	Co-design session
2: feature prioritization	13 research participants and 9 CAB members	Feature prioritization	Kano customer satisfaction survey
3: app design and esthetics	7	Wireframing and prototyping; determining the visual esthetic of the app	Participatory design and iteration; semistructured interview and idea generation
4: expert usability testing	2 (expert evaluators)	Feature usability testing	Heuristic evaluation: cognitive walk-through and System Usability Scale scores

#### Design Session 1: Feature Requirement Exploration

##### Overview

The first design session was exploratory and represented stage 1 of the participatory design approach by Spinuzzi [55]. We asked participants to free list app features that they found satisfying and sort the features into thematic categories (eg, privacy and user interface). Free listing is a fast way to generate many ideas in a short period [71]. During the exercise, participants created sticky notes with desired features and then moved the sticky notes between quadrants, conferring and discussing their opinions with the other participants. We also conducted a value proposition exercise to understand end users’ expectations of using the TGHIR app [72]. We asked participants to consider the perspectives of other potential end users using a templated value proposition statement (ie, *For people who ___, this mobile app is ___ that will provide ___*).

##### Results: Design Session 1

**Figure 4:**
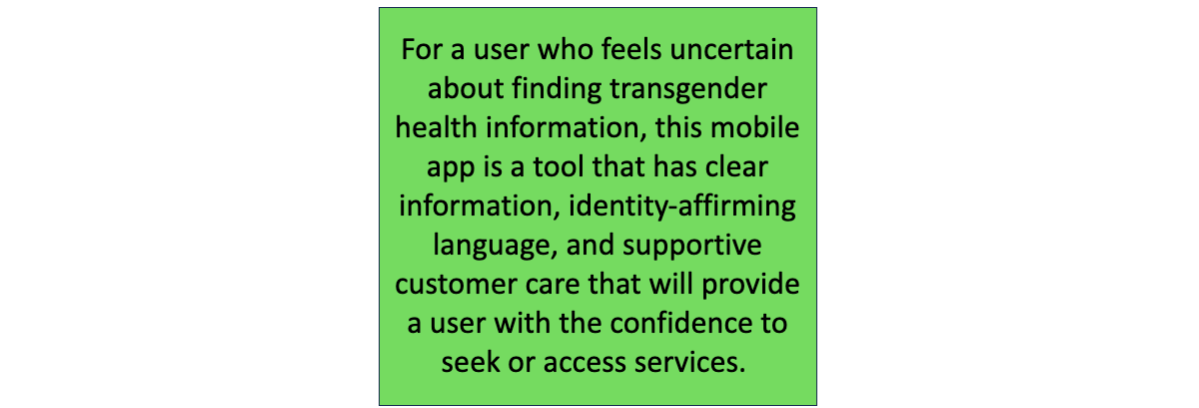
Sample feature value proposition statement from design session 1.

A total of 4 participants generated 35 potential features and 14 value propositions. The research team extrapolated additional features from the value propositions. Figure 4 shows an example of a value proposition. The research team then grouped similar features and removed any duplicates to generate a final list of 23 features (Textbox 1), which was used as input for design session 2.

**Features classified using the Kano Model of Customer Satisfaction.**

***Must-be* (requirements that a user expects to be implemented and that the user would be dissatisfied if they were not available)**

- External links to resources in general and links to hormone therapy mentioned specifically (external resources)

***One-dimensional* (requirements are related to the quality of a feature or service such that greater quality is correlated with greater satisfaction)**

- Search by topic and subcategory functionality (user interface)
- External links to mental health services (external resources)
- External links to community services (external resources)
- *Contact us* functionality (reviews)
- User interface simplicity (user interface)
- Account settings (user interface)
- Use of in-app pronouns tailored to the end user (pronouns)
- Remembering user settings (user interface/privacy)
- Culturally relevant language (user interface)
- Accessibility options (user interface)
- Removal of inappropriate posts (reviews)
- No data sharing with third parties (privacy)
- Protection of credentials for logging in (privacy)
- Data security (privacy)

***Attractive* (requirements may not be expected or expressed by a user but would make them satisfied if they were implemented)**

- Search by typing (interface)
- Ability of users to suggest new information sources (user interface/reviews)

***Indifferent* (a user has a neutral opinion on whether a feature is implemented)**

- History of viewed content (user interface)
- External link to pronoun tester (pronouns)
- Gender identity filter to support searching resources (user interface)
- Resource synopsis (external resources)
- Review of resource information credibility (user interface)
- News feed (user interface)

#### Design Session 2: Feature Prioritization

##### Overview

In design session 2, stage 2 (discovery) of the participatory design model by Spinuzzi [55], we asked participants to prioritize the 23 app features identified in design session 1 [55] using the Kano Model of Customer Satisfaction exercise [73,74]. The Kano Model classifies features as must-be, one-dimensional, attractive, and indifferent (Textbox 1). The Kano survey was completed via REDCap (Research Electronic Data Capture; Vanderbilt University). To allow the research team to better understand the justification for feature prioritization, several participants took part in a web-based facilitated exercise to prioritize features after independently completing the Kano survey. The web-based session allowed participants to use a bullseye visualization to prioritize features and prompted discussion about their choices.

##### Results: Design Session 2

A total of 22 participants, including 9 CAB members, completed the Kano survey. Textbox 1 lists the features in order of prioritization according to the 22 Kano survey responses.

**Figure 5:**
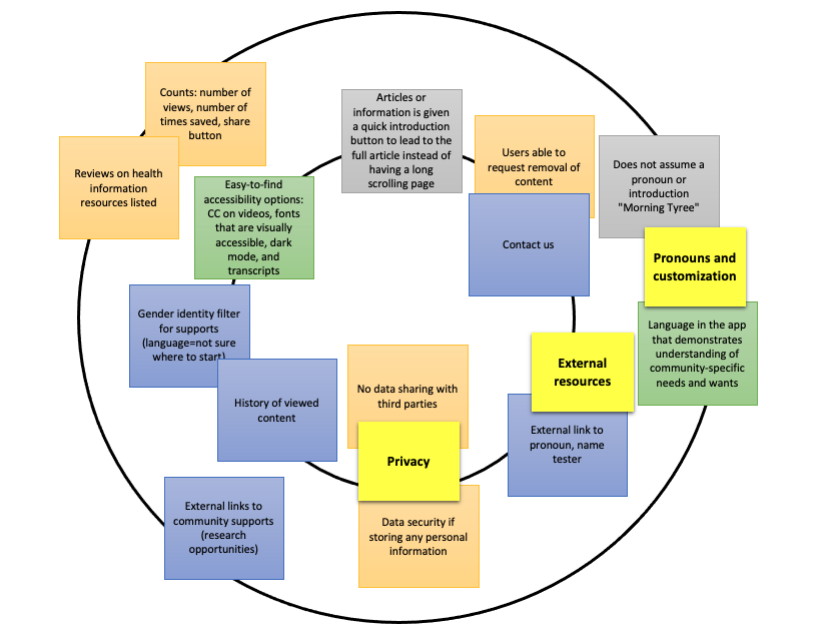
Bullseye prioritization exercise. Recreation of the 2 innermost rings from the original image to enhance readability. CC: closed captioning.

The bullseye exercise resulted in the image shown in Figure 5. Owing to the interactive nature of this activity, participants only discussed and arranged 65% (15/23) of the features. We compared the prioritization from the bullseye exercise with the classification from the Kano survey. The features in the highest-priority center of the bullseyes were *links to references*, *no data sharing with 3rd parties* (eg, not selling personal information), and “contact us,” all of which were classified as must-be or one-dimensional in the Kano results. The multilingual option and compatibility with assistive technology features were both in the center of the bullseye and also ranked as *One-Dimensional*; unfortunately, these were out of scope for this project’s funding.

#### Design Session 3: App Design

##### Overview

**Figure 6:**
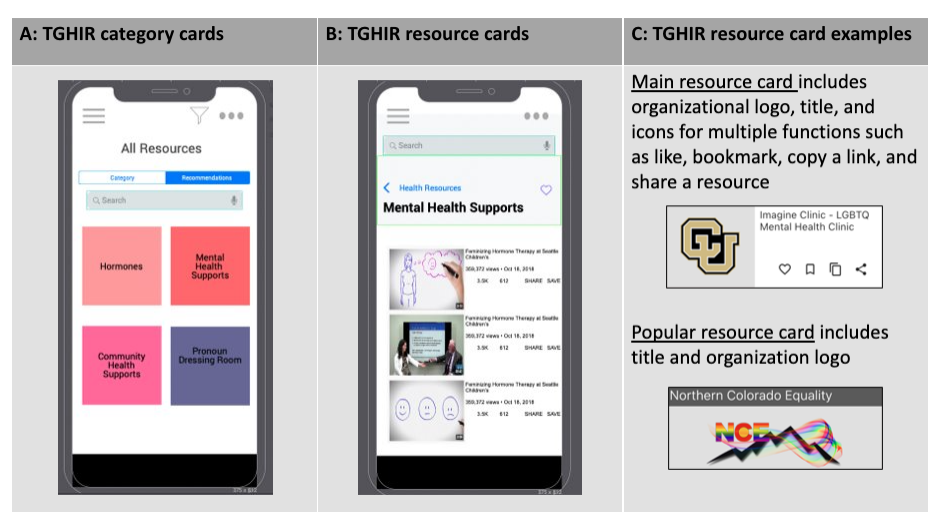
Midfidelity mock-up screens—(A) category cards and (B) resource cards—and final user interface—(C) resource cards. (A) is a mock-up screen with a list of category cards, (B) is a mock-up screen with a list of resource cards for a selected category, and (C) is the final prototype of the 2 types of resource cards—one to display information of a single resource within a category and one to display popular resources among the most viewed, liked, or bookmarked. TGHIR: transgender health information resource.

Design session 3, stage 3 (prototyping) of the participatory design model by Spinuzzi [55], was the wireframing and prototyping of the TGHIR app features. In this session, participants iteratively envisioned and informed the various features of the TGHIR app using midfidelity mock-up screens (Figures 6A and 6B). The prototypes were created by the design session moderator using the Justinmind prototyping tool [70]. We held 2 sessions covering the account creation process, the menu page, and the main information resource screens. We also shared an early prototype with the CAB to collect insights and feedback.

##### Results: Design Session 3—App Design

The 9 participants felt that all TGHIR app design elements should support easy access to the TGHIR resources and quick assessment of credibility. Owing to concerns about privacy and potential misgendering raised by the design participants, the account settings and use of in-app pronouns were discarded from the final list of features. This decision resulted in no use of personal profiles with self-identified pronouns or identifiers such as names and birthdates. The only personal information used by the system was the email address used to create a user account and log in to the system. However, after logging in, only the autogenerated hash ID would be used to record user activities, such as indicating resource *likes* and bookmarking resources.

Participants also felt that it was important to visually communicate the credibility of the TGHIR resources by displaying the content creator’s logo. Participants desired that the TGHIR Resource Card display the title, a short description, and a leading image and provide a preview of the resource, allowing them to choose whether to access it. The final version of each type of card varied slightly from the midfidelity examples. For instance, Figure 6C shows the final and enhanced design of the resource cards. Finally, some participants proposed changing the TGHIR app icon to a non–TGD-associated image such as a calendar.

CAB feedback also contributed to the revision and redesign of the app features. For instance, an earlier prototype forced users to immediately *label* themselves as either transmasculine or transfeminine upon entering the app in response to earlier input where users only wanted to see information relevant to their gender identity. This was ultimately deemed more harmful than helpful because of the breadth of gender identity diversity within the TGD community.

#### Design Session 3: Esthetics

##### Overview

One participant, a student of graphic art and user design, engaged in a 1:1 design session to provide input on the TGHIR app esthetics. The design session was conducted as a semistructured interview, and the participant was asked to provide their opinions and suggestions regarding the app’s user interface and its potential impact on user experience. They contributed to five topics related to esthetic appeal: (1) color palette, (2) font type, (3) name of the TGHIR app, (4) TGHIR app logo, and (5) use of backgrounds and images.

##### Results: Design Session 3—Aesthetics

This design session generated important feedback and insights to inform the final appearance of the app layout, color, and font scheme. The participant suggested blue hues for background, cards, and buttons (eg, “blue is a color that feels relaxed”) but recommended that we not use the Flutter (Google) default blue for the header and foot bar as it was similar to Facebook’s blue hue. Thus, to avoid similarities or confusion with commonly used social apps, we selected yellow for the app’s header and footer. It was also recommended not to use any background images or animations because of potential loading performance issues and user frustration.

**Figure 7:**
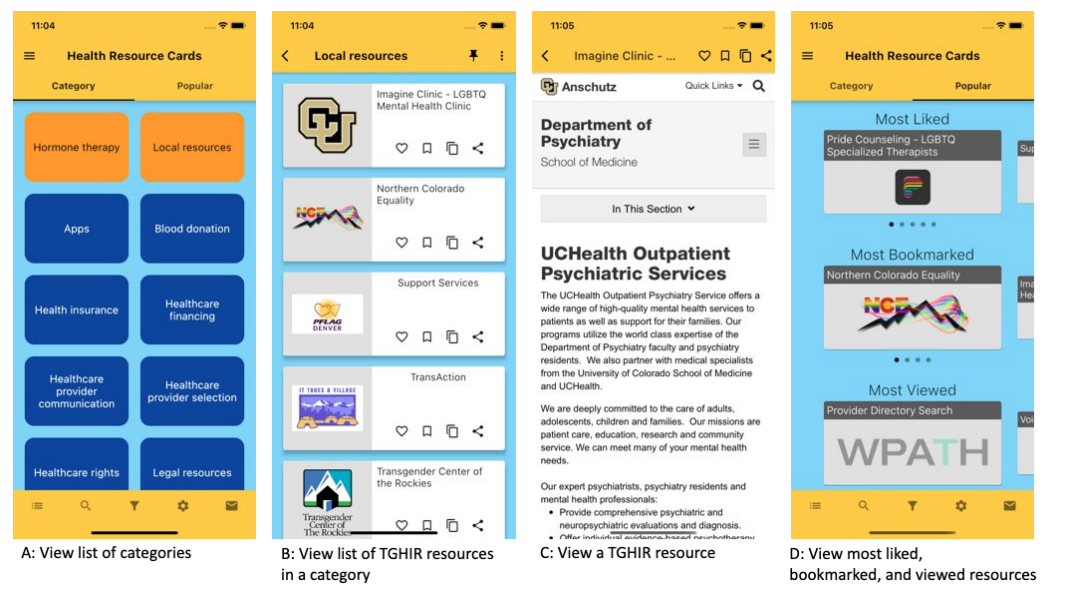
Screenshots of the main transgender health information resource (TGHIR) app features.

Another suggestion was “making timely resources pop out on the category page so people can see it quickly if they need it.” This suggestion led to the creation of the *categories of interest* feature, where end users could select their preferred categories of interest and the TGHIR app pinned the categories to the top of the *Health Resource Cards* page using a different background color to indicate pinned categories. For instance, selecting the categories *hormone therapy* and *local resources* turned the blue category cards into orange cards (see the example in Figure 7A). This option would allow users to have easy access to preferred categories. The TGHIR app offered two ways for end users to select preferred categories: (1) by clicking on the shortcut icon in the footer (first icon on the bottom left; Figure 7A) to open the *categories of interest* page where checkboxes can be used to indicate a preference and (2) by clicking on the pushpin icon that is available when viewing the list of resources in a specific category (icon on the right of the category title; Figure 7B). An overview of the app is provided in Figures 7A, 7B, 7C, and 7D. The app layout has 3 main areas. The first is the header, which includes the title of the page being displayed; the overall menu (hamburger menu icon; A and D); icons for features such as returning to the previous screen function (back icon; B and C) and marking the current category as preferred (pushpin icon; B); and icons to like, bookmark, copy, and share resources (C). The second area is the body, which is used mainly to display the list of category cards (A), the list of resource cards (B), the content of the resource (C), and the list of popular resources (D). The third area is the footer, which displays the icons to access features such as category selection, search, filter, settings, and *contact us*.

### Phase 3: Mobile App 1.0 Build and Release

#### App Development

In this phase, we reviewed the features and prototypes defined in the previous phase and translated them into requirements for developing the TGHIR app. We used Flutter [75], an open-source user interface development kit by Google, to develop a cross-platform mobile app for both Android and iOS devices. The database uses Firebase Firestore (Firebase Inc) [76], a NoSQL cloud database; the search engine uses Amazon OpenSearch Service (Amazon Web Services) [77]; and the email system uses SendGrid [78], a communication platform for email transactions.

We adopted the Agile software development methodology [79-81], an effective and efficient method to develop software that uses an iterative approach, incremental development, continuous value delivery, and user feedback [82]. On the basis of the features implemented in a sprint (2-week development cycle), the development team performed functional, interface, performance, system, service, and security testing of the TGHIR app on both Android and iOS devices. Examples of screens with the main features developed are shown in Figure 7.

#### Search Engine Development

With the goal of enhancing access to relevant TGD health information, we created a search engine that indexed information from the librarian, identified TGHIR resource pages, and linked subpages within the same domain. The URLs for the list of TGHIR resources were used as seed pages for the search engine. The search feature in the app allowed a user to type a search term and receive in return a list of pages that contained the searched term. Users could use wildcards to search for alternate spellings and variations on a root word.

#### Design Session 4: Expert Usability Testing

##### Overview

During this phase, we conducted design session 4 (expert usability testing), which provided insights and feedback to revise the app features with the goal of enhancing usability. This session consisted of experienced health information technologists, referred to as expert evaluators, with backgrounds in app design and integration. The TGHIR app version 1.0 was uploaded to the Google Play Store and the Apple Store Connect (TestFlight) so that the expert evaluators could install and test the app during the session. Each of the experts completed a cognitive walk-through [83] and was asked to complete 10 tasks associated with the TGHIR app features to identify any usability problems. Expert evaluators independently responded to item 3 on the System Usability Scale (SUS) [84,85], “I thought the feature was easy to use,” for each of the 10 app features/tasks (Table 2). We selected this single item to minimize participant response burden and optimize face validity.

**Table 2:** Feature and task evaluation by experts during phase 3 and by test users during phase 4.

Feature/task evaluation	Phase 3: design session 4 (expert evaluators; n=2)—SUS^a^ item 3				Phase 4: usability and quality evaluation (test users; n=13)—SUS items 3 and 8	
	Strongly disagree/disagree, n	Neutral, n	Strongly agree/agree, n	Estimated SUS numeric score		Category score
Create an account	0	0	2	78.42		Good
Select preferred categories	2	0	0	77.74		Good
Find a specific item using the category cards	0	0	2	76.95		Good
Use filter to narrow resources	1	0	1	65.37		Okay
Like a resource	0	0	2	80.01		Good
Bookmark a resource	0	0	2	76.09		Good
Search to find a specific resource	0	1	1	68.46		Good
Send a message to developers	0	0	2	82.34		Excellent
Share a new resource for the community with the developers	0	0	2	79.25		Good
Find the most liked resource	0	0	2	85.42		Excellent

^a^SUS: System Usability Scale.

##### Results: Design Session 4

The results from our 2 expert evaluators showed that most app features were easy to use (Table 2). The selection of preferred categories, feature 2, was not perceived as easy to use by either expert evaluator. Neither expert evaluator saw the option of selecting a category of interest as obvious or intuitive; the menu option was buried, and the pushpin option was overlooked. Owing to the comments on difficulty finding and accessing some features, the development team created the footer with shortcut icons to facilitate access to the important features, such as category selection, search, filter, settings, and *contact us* interface.

One expert evaluator was concerned that the filter (feature 4) was difficult to use and end users would find it difficult to understand how the filter feature returned results. Specifically, the filter was built to facilitate searching for resources across multiple domains and categories. For instance, a user can select a broad domain, such as legal and financial topics, and be presented with categories that have information related to the selected domain (ie, health insurance, health care financing, health care rights, and legal resources). The filter also suggests broad categories based on a user’s narrow input. For example, a user selecting the narrow topic *health insurance* would be reminded, based on filtering, that the app has insurance resources related to legal and financial topics. This approach can help users learn about different facets of the transition process.

### Phase 4: Final Assessment—Usability and Quality Evaluation

In phase 4, test users installed and used the released TGHIR mobile app on their smartphones for a period of 4 weeks and participated in 3 evaluations. First, we conducted cognitive walk-through interviews within 2 days of the TGHIR app being installed on the test users’ phones to understand the performance of all the features. Test users were asked to try the same 10 features/tasks that the experts had evaluated (Table 2). To assess feature usability, test users were asked to respond to items 3 and 8 on the SUS [85]—*I thought the feature was easy to* use and *I found the feature very cumbersome/awkward to* use, respectively [86]—for each of the 10 features via a REDCap survey. The SUS is unidimensional and only measures 1 construct, that is, perceived usability. It has been shown that collecting responses to items 3 and 8 is 96% accurate in assessing system usability while also decreasing participant burden [86].

Test users completed the user version of the Mobile App Rating Scale (uMARS) via REDCap at 2 and 4 weeks after app installation (T1 and T2, respectively). The uMARS is a widely used scale for evaluating the quality of mobile health (mHealth) apps [87]. The uMARS is a modified version of the Mobile App Rating Scale (MARS) that is designed to be used by end users of a mobile app without training or expertise in mHealth technology or in the related health field. The uMARS contains 16 items assessing 4 dimensions of objective quality—engagement, functionality, esthetics, and information—and includes 4 items for the dimension of subjective quality. We also used 3 optional items for the dimension of perceived impact. All uMARS items are assessed on a 5-point scale (1=inadequate, 2=poor, 3=acceptable, 4=good, and 5=excellent) [87]. The MARS and uMARS have been shown to have excellent internal consistency, with high individual Cronbach α values for all subscales, and are valid, reliable, and accurate in measuring health app quality [87,88]. The MARS scores have also been shown to correlate with mHealth app revenue, monthly active users, and user downloads [89].

## Results

A total of 13 test users participated in the final usability and quality assessment of the TGHIR app version 1.0. The SUS assessment showed good to excellent usability for all features except the *Use filter to narrow resources* (Table 2). The cognitive walk-through interview results showed that the TGHIR app had high usability overall. Test user TGU104 said the following:

I love this app. My main feedback that I see growth in is a tutorial that does an overview...this is groundbreaking for there to be one place to actually find these links.

Once this test user realized that the TGHIR resources were credible health resources, they were excited and spoke highly of the TGHIR app. Another test user, TGU105, said the following:

I really like the way the cards look, and obviously I like the way the search function works, but you may want to find a way to integrate them.

In general, most of the feedback received was positive, with suggestions on how to make tweaks for increased usability. Users indicated that the filter worked and was relatively easy to find within the app (owing to the recognizable filter icon), but there was some confusion regarding the labeled parts of the feature (broad domains and narrow topics). A test user suggested that we add filters that would work to separate TGHIR resources by gender identity. Note that, upon sharing an early prototype of the TGHIR app with the CAB, the CAB raised concerns about a feature that required users to first select a gender identity, which the CAB felt might inappropriately force users to put themselves into a predefined category. Therefore, this feature was ultimately not incorporated into the app.

The results of the uMARS assessment are presented in Table 3. The overall mean uMARS objective quality score at the first evaluation (T1) was 4.13 (SD 0.29), indicating *good* overall TGHIR app quality. At the second evaluation (T2), the overall mean uMARS objective quality score increased to 4.25 (SD 0.35), indicating *good* overall TGHIR app quality. It appears that with continued use of the TGHIR app, test users perceived the app to be of better quality. The subjective quality rating was 3.75 (SD 0.83), and the perceived impact rating was 4.45 (SD 0.40). The information rating at T2 was 4.75 (SD 0.16), which is near *excellent.*

**Table 3:** Transgender health information resource user testing (T1 and T2) results of the user version of the Mobile App Rating Scale (uMARS).

uMARS domain and subcategory			T1 (n=13), mean (SD)		T1 mean score assessment^a^		T2 (n=13), mean (SD)		T2 mean score assessment^a^		Mean score difference (T2 – T1)
**Objective quality subscale scores**											
	Engagement	3.80 (0.69)		Acceptable		3.98 (0.69)		Acceptable		0.18	
	Functionality	4.12 (0.19)		Good		4.20 (0.11)		Good		0.08	
	Esthetics	4.08 (0.20)		Good		4.06 (0.14)		Good		−0.02	
	Information	4.50 (0.26)		Good		4.75 (0.16)		Good-excellent		0.25	
Objective quality total mean scores			4.13 (0.29)		Good		4.25 (0.35)		Good		0.12
Subjective quality			3.66 (0.75)		Acceptable		3.75 (0.83)		Acceptable		0.09
Perceived impact			4.31 (0.42)		Acceptable		4.45 (0.40)		Good		0.14

^a^uMARS categories are assessed on a 5-point scale: 1=inadequate, 2=poor, 3=acceptable, 4=good, and 5=excellent.

## Discussion

### Principal Findings

Through the process of participatory design [55], we successfully cocreated a TGHIR delivered as a mobile app for and with people who are TGD to provide access to credible health information resources. The participatory design process was guided by our conceptual framework, the multiphase methodology by Spinuzzi [55], and principles of community engagement. A partnership with the CAB and insights from research participants confirmed the TGD community’s need for accessible and credible health information. It also illustrated their frustrations and barriers to obtaining credible health information. Barriers to obtaining credible information include a lack of knowledge by clinicians; the lack of information on the internet and the inability to find and identify it even when it does exist; and, finally, the lack of empirical evidence for many of their questions. For the TGHIR app to be of value, it had to achieve 2 goals. First, the app should direct people to credible, useful information, and second, the app should be easy to use and satisfying to the user. After 4 weeks of use, users gave the TGHIR app an overall objective quality rating of 4.25, a subjective quality rating of 3.75, and a perceived impact rating of 4.45 out of 5.00 on the uMARS scale, which equates to a *good*-quality mobile app. Most importantly, the information category had the highest uMARS score of 4.75 out of 5.00. The uMARS scores indicate our success with both goals: a usable app and valuable information. The overall quality rating improved during the 2 weeks of use, suggesting that users gained familiarity with the app features. Mobile apps that have overall uMARS ratings of *good* quality are more likely to be adopted by the intended users than poor-quality apps [87,89]. Our 4-week engagement score was 3.98, and although this did increase from week 2 to week 4, it is not clear how this score would have changed over a longer time period. A review of publicly available asthma apps found that app quality varied and that engagement was often the lowest-scoring dimension [90]. Our TGHIR app scores were better than the asthma app scores that had average objective and subjective quality scores of 3.17 (range 1.54-4.55) and 2.65 (range 1.00-4.50), respectively. Our scorers were also the end users themselves, which may be better correlated with end-user use.

The intensity of engagement is interesting to consider for mHealth apps that seek to change user behavior, such as apps to help patients manage chronic diseases, where long-term and frequent engagement may be critical to improve outcomes [91,92]. Long-term engagement with diabetes management apps has been limited [93], and experts have suggested that virtual coaching [94] along with wellness and chronic disease app use may enhance engagement and outcomes. A recent paper exploring the value of mHealth apps for patients and how they can be incorporated into traditional medical care delivery first classified apps into four categories: (1) aiding diagnosis or decision-making, (2) improving outcomes through better disease management, (3) stand-alone digital therapeutic devices, and (4) primarily delivering education. [95] The TGHIR app falls best into category 4, as it was designed to provide information to the TGD community, and in that sense, it may assist with decision-making (ie, *Should I go on testosterone or not?*) and condition self-management but only by providing information and improving one’s knowledge. Information is essential for education, but we did not create specific educational materials, although some resources available through our app may have been developed with educational intent, nor was the TGHIR app a diagnostic or decision support app for a specific concern, such as whether a skin lesion is cancerous or to provide triage advice.

A recent randomized controlled trial of an mHealth app designed to deliver information to educate patients regarding knee pain showed that it increased a patient’s disease-related knowledge [96]. Others have also suggested that informational/educational apps may lead to improved processes and outcomes for diseases and conditions such as heart failure, inflammatory bowel disease, recent tonsillectomy, and various cancers [97-99]. Others have proposed that informational/educational apps may lead to improved patient-provider communications and improved decision-making, which is something that we would like to assess in future research.

The TGHIR app benefited from a research team that included clinicians who were experts in evidence-based transgender health care and a medical librarian who could search, identify, and deem credible the information resources made available via the app. Manually maintaining credible health information is a resource-intensive endeavor. Automated methods to find information on both peer-reviewed medical manuscripts and non–peer-reviewed materials would be valuable, but this also needs to be paired by credibility assessments and methods to tag content so that the TGHIR content can be searched for and found by users. Community crowdsourcing to create useful and credible health information resources has been found to have more reliability when professionals or experts are present in the process of content creation. Many concerns exist regarding the expertise (ie, no medical or health education expertise) and intent of the authors (ie, want to sell a product) [100,101]. Whether community users can judge credibility is also debated. There is research suggesting that people often report judging source credibility, but observational studies suggest otherwise [102-104]. Owing to concerns expressed with finding credible information and concerns about author intentions, we did create a *contact us* feature allowing users to recommend additional resources and removal of content deemed noncredible or offensive. We built a dedicated PubMed or MEDLINE search for use with the MedlinePlus application programming interface [105] and a search feature for ClinicalTrials.gov, but the prebuilt searches were not incorporated into the final TGHIR app because of time constraints. A link to the “Trust It or Trash It” resource [68] was also not incorporated into the prototype app. Both are planned for future versions pending additional funding.

There is a clear need and value proposition for the TGHIR app. The information provided by the app is not intended to replace competent clinical TGD health care and is intended to assist laypersons so they have the information needed to be engaged and informed participants in their health and well-being.

Some participatory methodological insights were generated during the project and may be helpful to other researchers. Our perception was that the TGD participants were grateful for the opportunity to take part and be involved in something that was being designed for them and with them. Several participants went beyond our requested involvement to offer advice on graphic design and other areas in which they had expertise.

### Limitations and Strengths

Limitations included a focus on users based in Colorado and challenges in recruiting and, thus, co-designing with racially and ethnically diverse individuals. Our medical librarian did identify health information resources in Spanish, and our back-end health information resource database is designed to store the language of the resource, but we had limited resources for providing the TGHIR app interface in languages other than English and providing access to non–English-language resources. Despite striving to recruit demographically diverse co-design and end-user testing participants, we had difficulty recruiting TGD people of color and from rural settings. Although 34% (11/32) of our participants indicated that they were Black or African American, Asian, Native American, and of multiple races, we were not able to recruit any Black or African American test users. We can only hypothesize why this was difficult, including reasons such as Colorado’s demographics and the multitude of political issues necessitating this community to be active advocates at the time of this project. We expect that the TGHIR app would be useful to people of color and from rural settings regarding the user interface and functions, but it remains a question as to whether the information would have been rated as highly by these underrepresented groups. Some of the information resources were local TGD resources, which were more likely to be in the Denver metropolitan area and, therefore, might be deemed less valuable to those residing outside the metropolitan area. It is also true that rural areas are less likely to have the same extent of LGBTQ+ resources as urban areas, which is not a fault of the TGHIR app. The age distribution of our participants, 18 to ≥50 years, is a strength of this study.

### Conclusions

Using methods of participatory design with the TGD community and in partnership with a CAB, we were able to co-design and develop a health information resource delivered via a mobile app for persons who are TGD and their care partners. Users felt that this app would be beneficial to them and that it provided needed information. A health information app is only as good as the information it makes accessible, and ongoing updating and maintenance of information resources in any information app is a challenge. Next steps include work to automate/semiautomate methods to identify relevant and credible information and testing for clinical effectiveness, including outcomes such as more engaged and useful interactions with health care providers and being better informed of the options available and their risks and benefits to support informed decision-making.

## Appendix

Multimedia Appendix 1 Transgender health information resources.

Multimedia Appendix 2 Transgender health information resources app credibility resources.

Multimedia Appendix 3 Transgender health information resources health information content.

## Abbreviations

CAB: Community Advisory Board
FG: focus group
HIT: health IT
LGBTQ: lesbian, gay, bisexual, transgender, queer or questioning
MARS: Mobile App Rating Scale
mHealth: mobile health
REDCap: Research Electronic Data Capture
SUS: System Usability Scale
TGD: transgender and gender diverse
TGHIR: transgender health information resource
uMARS: user version of the Mobile App Rating Scale
