# Supplementary material for: A Transgender Health Information Resource: Participatory Design Study
Source: JMIR Hum Factors. 2023 Jun 15;10:e42382. doi: 10.2196/42382 (PMC10337357; doi:10.2196/42382)
Supplement: Multimedia Appendix 1 [file humanfactors_v10i1e42382_app1.pdf]

TGHIR app Content Search Terms

Transgender identity terms:

- "gender affirmation"|"gender confirmation"|genderqueer|"gender questioning"|"gender transitioning"|nonbinary|transgender|transfemale|transfeminine|transwoman|transwomen|transmale|transmasculine|transman|transmen

Healthcare terms:

- "hormone therapy"|"hormone replacement therapy"|hormones
- surgery
- "primary|preventive|preventative care"|"prostate exam"|mammogram|"breast exam"|"pap smear"|"pelvic exam"|"prostate|breast|cervical|ovarian cancer"
- "health insurance"|Medicaid|Medicare
- "birth control"|contraception|contraceptives
- menstruation|"menstrual cycle"
- pregnant|pregnancy|fertility|reproduction|infertility
- breastfeeding|"breast feeding"|"breast milk"|"chest feeding"|lactation
- "blood donation|donors|bank"

Transgender identity terms were searched in combination with healthcare terms. The pipe symbol (|) was used in place of the word "OR".
