# Supplementary material for: A Transgender Health Information Resource: Participatory Design Study
Source: JMIR Hum Factors. 2023 Jun 15;10:e42382. doi: 10.2196/42382 (PMC10337357; doi:10.2196/42382)
Supplement: Multimedia Appendix 2 [file humanfactors_v10i1e42382_app2.pdf]

TGHIR app Credibility Resources

The following resources were used to identify principles of credibility (such as authority, bias, currency, funding, privacy, and quality) which were used to guide resource selection:

- DISCERN (http://www.discern.org.uk/)
- QUEST, QUality Evaluation Scoring Tool (https://www.ncbi.nlm.nih.gov/pmc/articles/PMC6194721/)
- HONcode, Health On the Net Foundation Code of Conduct (https://www.hon.ch/HONcode/)
- Health Website Evaluation Tool (https://www.hon.ch/HONcode/Patients/HealthEvaluationTool.html)
- Trust It or Trash It? (http://www.trustortrash.org/)
- Evaluating Internet Health Information: A Tutorial from the National Library of Medicine (https://medlineplus.gov/webeval/webeval.html).
